# Unilateral percutaneous vertebroplasty for osteoporotic lumbar compression fractures: a comparative study between transverse process root-pedicle approach and conventional transpedicular approach

**DOI:** 10.1186/s13018-021-02219-6

**Published:** 2021-01-21

**Authors:** Wenwu Zhang, Shenpeng Liu, Xianhua Liu, Xiang Li, Le Wang, Yong Wan

**Affiliations:** 1 Department of Spine Surgery, The First Affiliated Hospital of Sun Yat-sen University, No. 58, Zhongshan 2nd Road, Yuexiu District, Guangzhou, 510080 Guangdong China; 2 Department of Orthopedic, The First Affiliated Hospital of Xinxiang Medical University, No. 88, Jiankang Road, Weihui, 453100 Henan China; 3 Department of Psychiatry, The Second Affiliated Hospital of Xinxiang Medical University, No. 388, Jianshe Road, Xinxiang, 453002 Henan China

**Keywords:** Osteoporosis, Percutaneous vertebroplasty, Unilateral puncture, Osteoporotic lumbar compression fracture

## Abstract

**Purpose:**

Percutaneous vertebroplasty (PVP) is a routine operation for the treatment of osteoporotic lumbar compression fractures (OLCFs). As is well known, unilateral puncture is a common method. However, with the conventional transpedicular approach (CTPA), the cement may be asymmetrically dispersed, so some surgeons use the transverse process root-pedicle approach (TPRPA). The objective of this study was to compare the clinical results and bone cement distribution of PVP for OLCF with unilateral TPRPA and CTPA to identify the advantages and disadvantages of the two surgical options.

**Patients and methods:**

From January 2016 to June 2019, seventy-two elderly patients who underwent unilateral PVP for single-level OLCF were retrospectively reviewed. Operation time, injection amount and type of bone cement distribution, and bone cement leakage and surgical complications were recorded. The visual analog scale (VAS) scores and Oswestry disability index (ODI) scores were used to evaluate the clinical results. All patients were followed up for more than 12 months, and the assessment was based primarily on clinical and radiological outcomes.

**Results:**

There were significant differences in the surgical time and the volume and the type of bone cement distribution and the lost of operative vertebra height between the two groups. However, there was no significant difference in bone cement leakage. Moreover, there were no significant differences in VAS and ODI between the two groups at 2 days and 12 months after the operation.

**Conclusions:**

Unilateral TPRPA and CTPA are practical and feasible methods in PVP for the treatment of OLCF, and they have similar clinical effects. However, TPRPA has the advantages of a better distribution of bone cement and a shorter operation time and a better maintenance effect of injured vertebra height, without increasing the rate of bone cement leakage.

## Introduction

Osteoporotic lumbar compression fracture (OLCF) is the most common form of clinical osteoporosis complications. The pain caused by the fracture significantly affects the patient’s quality of life [1]. Percutaneous vertebroplasty (PVP) is widely used to treat such fractures, which can rapidly maintain the strength of the injured vertebral body, effectively relieve pain, and improve the quality of life of the patients [2, 3].

A bone cement injection through the bilateral pedicle approach is a common method, which has a definite therapeutic effect and can also create a symmetrical distribution of bone cement [4]. However, a bilateral puncture requires a longer operative time and more X-ray exposure. In recent years, most studies have indicated that a unilateral puncture is equivalent to bilateral puncture PVP [5, 6]. The conventional transpedicular approach (CTPA) is the most commonly used puncture route. However, there is concern about the asymmetric diffusion of bone cement in the vertebral body after CTPA.

Some studies [7, 8] on anatomy and imaging have shown that the extrapedicular and transverse process root-pedicle approach (TPRPA) can be used for PVP, but individual and level selection is required. TPRPA penetrates the pedicle through the root of the transverse process and lateral part of the pedicle, which allows for a greater abduction angle and it is relatively safer [8]. To the best of our knowledge, although TPRPA has been used by some surgeons, no published literature on its clinical results has included a comparative study between TPRPA and CTPA. The objective of this study was to compare and analyze the clinical effect of PVP for OLCF with unilateral TPRPA and CTPA, to identify the advantages and disadvantages of the two surgical options.

## Materials and methods

### General information

We performed a retrospective analysis of patients suffering from single-level OLCF treated with unilateral PVP from January 2016 to June 2019. According to the following criteria, seventy-two patients were included in this study. The inclusion criteria included: ① age of 65 years or older; ② T score < − 2.5 in a bone mineral density (BMD) examination of the lumbar vertebral; ③ a single-level fracture in the lumbar vertebra (L1–L5); ④ preoperative collapse exceeding 15% of the height of the injured vertebra, but not more than two-thirds; ⑤ preoperative pain was over 5, measured by a visual analog score (VAS); and ⑥ bisphosphonates were used for anti-osteoporosis therapy after surgery. Exclusion criteria includes: ① a fracture due to secondary osteoporosis; ② failure to obtained informed consent; ③ the patient received coagulopathy; ④ a pathological fracture caused by a tumor or spine infection; ⑤ there was symptomatic nerve damage; and ⑥ patients with incomplete data. Patients were assigned to the TPRPA group (38 cases) or the CTPA group (34 cases).

### Surgical procedure

#### TPRPA group

**Fig. 1:**
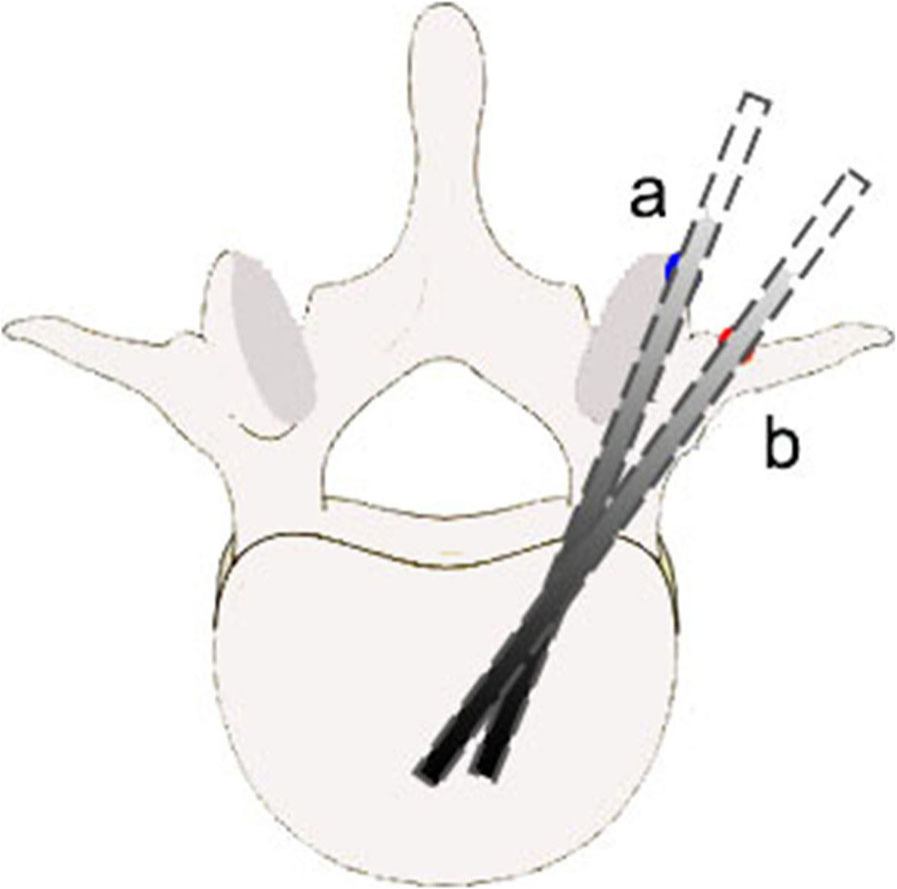
Schematic diagram of needle entry points of two methods. **a** CTPA group: the insertion point was located at the lateral facet of the articular process. **b** TPRPA group: the insertion point was located at the junction of the root of the transverse process and the pedicle

All of the PVP procedures were performed by two senior orthopedic surgeons (Wenwu Zhang and Shenpeng Liu) skilled in performing the procedures. Patients were placed in the prone position, and two soft pillows under the chest and pelvis were used to lift the abdomen to reduce any abdominal compression. Then, a manipulative reduction was performed to restore the height of the injured vertebra as much as possible. The target vertebral body was observed by a C-arm X-ray machine and we marked the body surface and the skin puncture point after fluoroscopy. Local infiltration of 1% lidocaine for anesthesia was applied, from the skin puncture point gradually deeper into the periosteum around the junction of the pedicle and the transverse process root. When the anesthesia was sufficient, a 0.5 cm incision was made at the skin puncture point, and the unilateral transverse process root–pedicle approach was adopted. The middle and upper part of the transverse process, and the junction area between the root of the transverse process and the lateral facet of the articular process, provided an effective puncture point. Fluoroscopy confirmed the entry point and determined the puncture direction at the same time (Fig. 1). The core puncture needle was inserted into the pedicle through the root of the transverse process under C-arm X-ray machine surveillance. The tilt angle of the puncture needle or tail was adjusted according to the intraoperative fluoroscopy and the angle measured by CT preoperatively. When the tip reached the posterior margin of the vertebral body, the point did not break through the medial wall of the pedicle under the anteroposterior position and lateral fluoroscopy and then continued to puncture to the first third of the vertebral body.

**Fig. 2:**
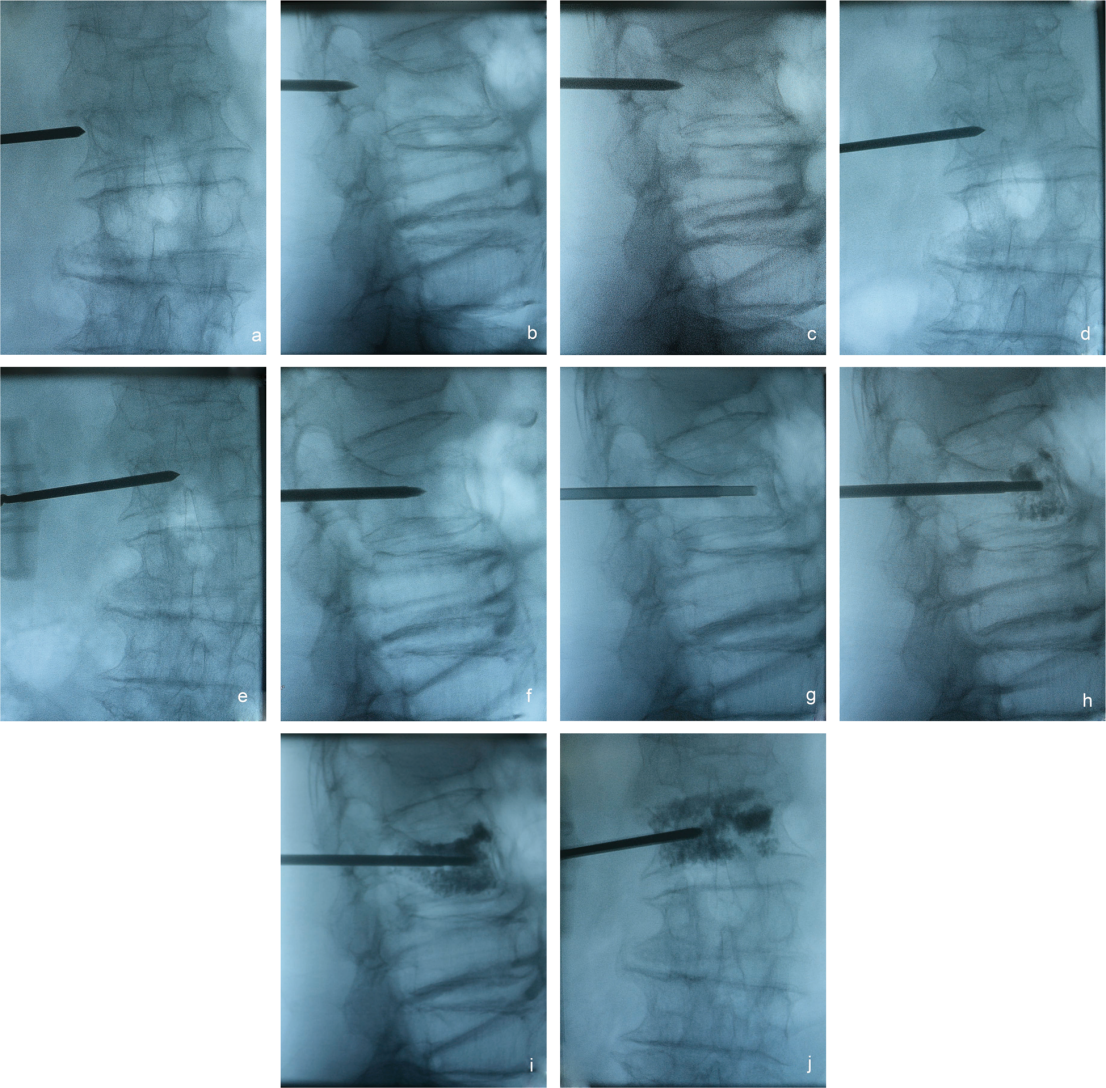
Intraoperative fluoroscopic images of the surgical procedure of the TPRPA PVP. **a** Posteroanterior fluoroscopy: the needle tip was located at the junction between the root of the transverse process and the pedicle. **b** Lateral fluoroscopy: the needle tip was approximately located one-third posterior to the pedicle. **c**, **d** Lateral and posteroanterior fluoroscopies: the cannula tip reached the posterior margin of the vertebral body and the inner wall of the pedicle. **e**, **f** Posteroanterior and lateral fluoroscopies; the needle tip reached the middle of the vertebral body. **g**, **h** Lateral fuoroscopies: the cannula reached the anterior part of the vertebral body and begun to inject bone cement. **i**, **j** Lateral and posteroanterior fluoroscopies: bone cement injection was complete

Patients were closely observed during the operation. The injection of bone cement was performed under the supervision of the C-arm. The injection was stopped when the bone cement reached the posterior third part of the vertebral body or when the leakage of bone cement occurred (Fig. 2). All injection components were removed after the injection of the bone cement, and the incision was sutured. All patients stayed in bed for 6 h and then resumed their normal activities the next day. Bisphosphonates were routinely used for anti-osteoporosis therapy on the second day after surgery.

#### CTPA group

The difference in the insertion point was the main difference between the two groups. The details are shown in Fig. 1. The CTPA insertion point was located at the 10 o’clock projection on the left pedicle and 2 o’clock projection on the right. Postoperative anti-osteoporosis treatment was the same as for the TPRPA group.

### Outcome measures

**Fig. 3:**
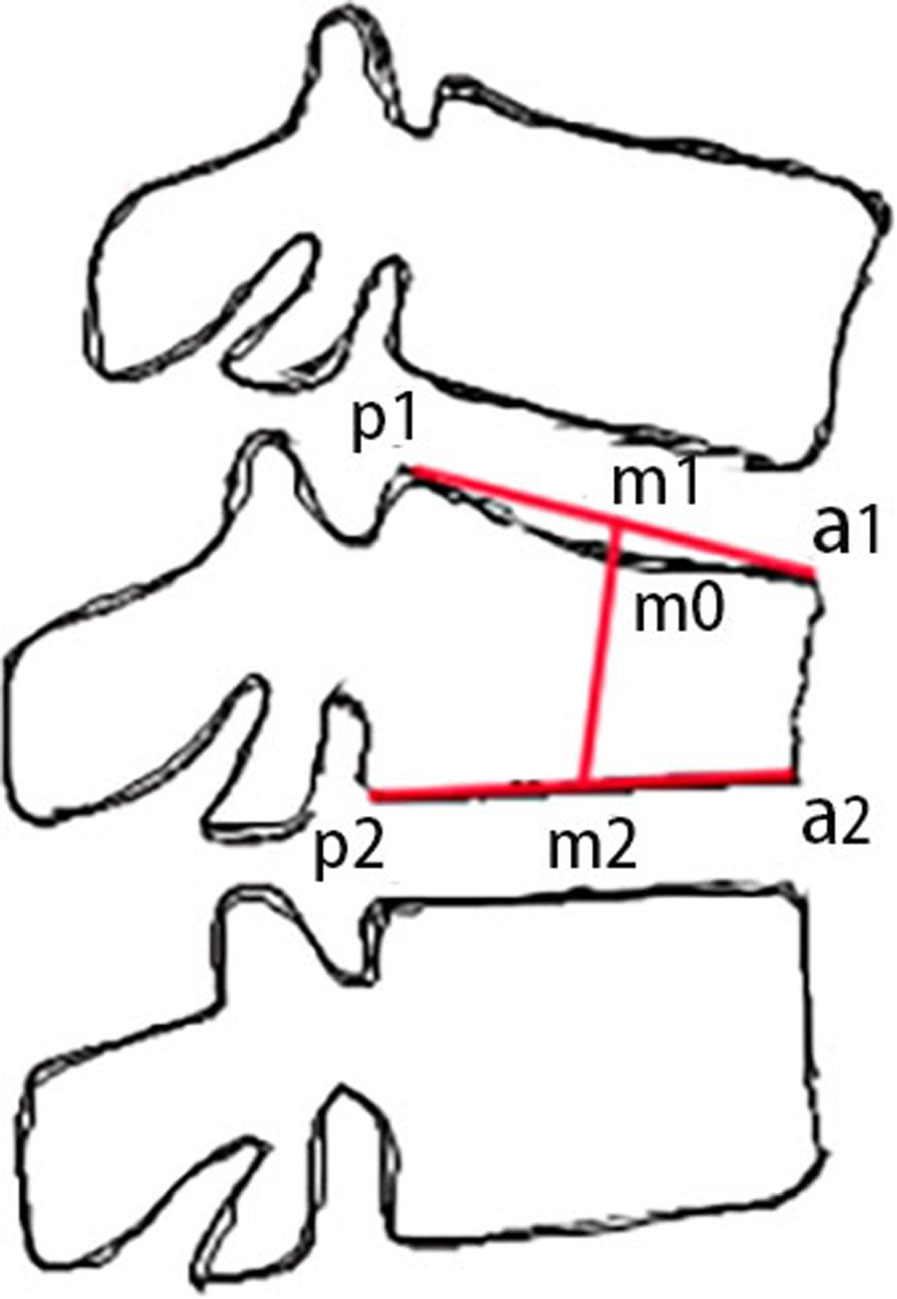
Method for measuring the height of injured vertebra

The operation time, bone cement leakage, injection amount, and distribution types of bone cement were recorded for each patient. The clinical assessments were evaluated by using the VAS and ODI preoperatively and at 2 days and 12 months postoperatively. Postoperative X-ray films were completed in all patients, and CT was further improved for suspected bone cement leakage. The distribution of bone cement was divided into two types according to the radiographs. Type 1 (T1): the bone cement contacted both the upper and lower endplates. Type 2 (T2): the bone cement missed at least one endplate. Detailed typing items could refer to the previous literature [9]. The height of the injured vertebra was measured on a lateral X-ray film and defined as follows: front edge of the upper endplate (a1), rear edge of the upper endplate (p1), the midpoint of the line between a1 and p1 (m1), front edge of the lower endplate (a2), rear edge of the lower endplate (p2), the midpoint of the line between a2 and p2 (m2), the point at which the line (m1m2) intersects the upper endplate (m0), and the height of the fractured vertebra (m0m2) (Fig. 3). The recovery height of injured vertebra was the difference between the height of the injured vertebra 2 days after the operation and the injured vertebra before the procedure. Loss of the height of the injured vertebra was the difference between 12 months after surgery and 2 days after surgery.

### Statistical assessments

All statistical analyses were performed with the SPSS software, version 22 (IBM, Armonk, USA). Paired *t* tests were used to compare pre-and postoperative variable outcome scores. Differences in the cement leakage rate and fracture site composition ratio of the 2 groups were assessed using the *χ*2 test, but the latter used a union treatment because some values were less than 5. *P* < 0.05 was considered to have statistical significance.

## Results

### Preoperative demographic characteristics and Outcomes

**Fig. 4:**
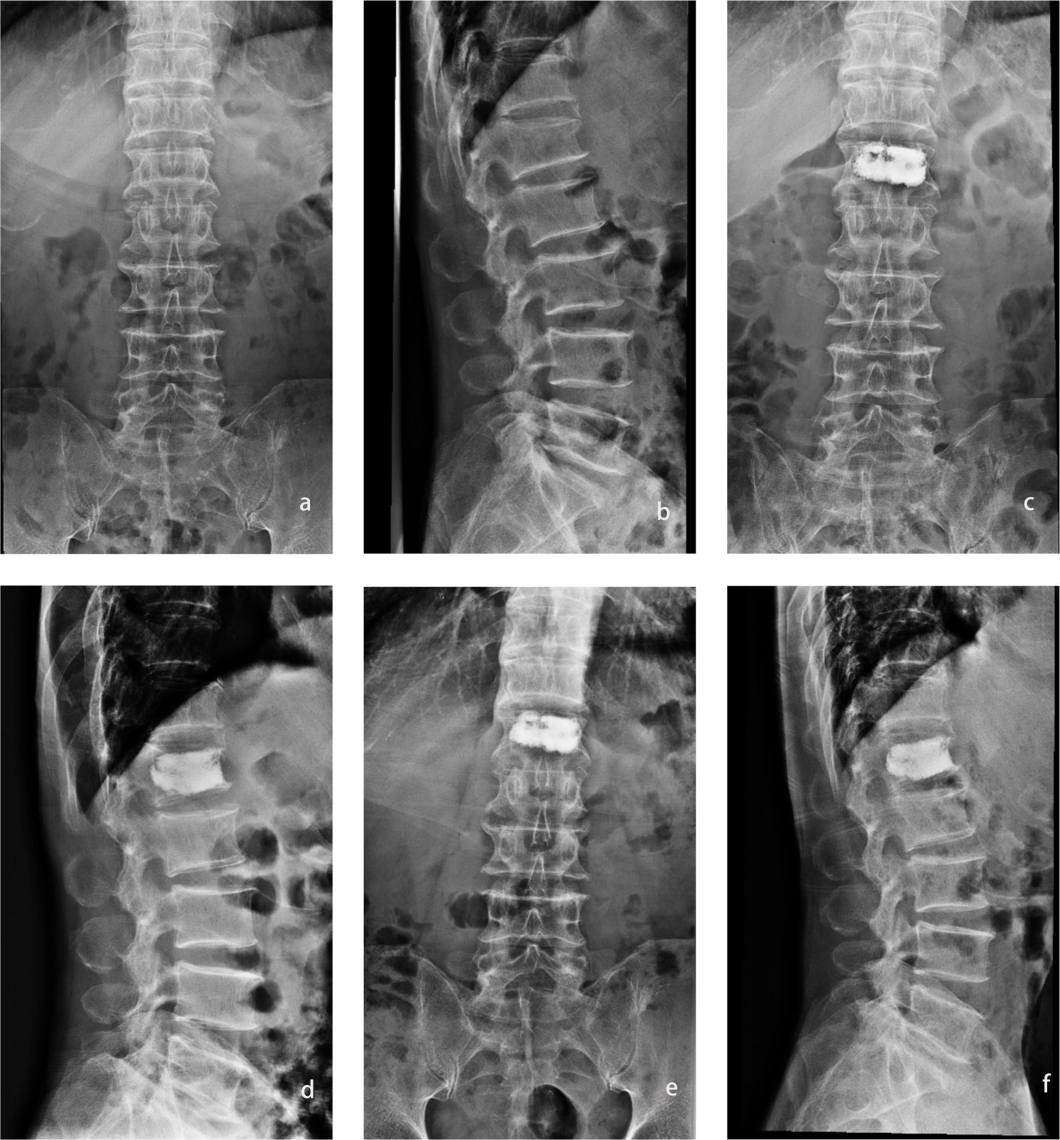
A 67-year-old man with L1 vertebra fracture treated with TPRPA PVP. **a**, **b** Preoperative spinal column: L1 vertebral fracture. **c**, **d** Two days postoperative, spinal column: the bone cement is symmetrically distributed and touches the upper and lower endplates. **e**, **f** One year after operation spinal column: the bone cement remains symmetrically distributed

The operations of both groups were completed successfully. All patients were followed for at least 12 months. No complications such as nerve damage or pedicle fracture occurred in all patients except for bone cement leakage. No recurrent fractures occurred during the 12 months of follow-up. In the baseline data of the patients, no significant difference was found between the two groups (Table 1). A typical case is shown in Fig. 4.

**Table 1:** Comparison of baseline data between group TPRPA and group CTPA

	Group TPRPA	Group CTPA	Statistics	*p*
Year	73.13 ± 7.15	72.35 ± 6.99	*t* = 0.466	0.643
Gender(M/F)	15/23	14/20	*χ*2 = 0.220	0.883
Body mass(kg)	73.53 ± 5.94	74.56 ± 5.00	*t* = -0.793	0.431
Height(cm)	167.37 ± 8.32	167.97 ± 5.76	*t* = -0.353	0.725
BMI(kg/m^2^)	26.27 ± 1.44	26.43 ± 1.99	*t* = − 0.501	0.618
OLCF level				
L1	20	18	*χ*2 = 0.001	0.979
L2	9	7		
L3	5	6		
L4	3	2		
L5	1	1

### Intraoperative measurement

For the average operation time and the volume of the injected cement, a statistically significant difference was found between the TPRPA and CTPA groups (*P* < 0.05). The operation time in the TPRPA group was significantly shorter than that in the CTPA group, and the bone cement injection amount was larger (Table 2).

**Table 2:** Comparison of two groups of the surgical time and bone cement injection volume

	Operation time (min)	Bone cement volume (ml)
Group TPRPA	50.53 ± 8.45	5.54 ± 0.72
Group CTPA	60.88 ± 11.96	4.62 ± 0.86
Statistics	*t* = − 4.277	*t* = 4.941
*P*	0.000	0.000

### Clinical results

There were no statistically significant differences in the VAS and ODI between the two groups. The scores of both groups were reduced after PVP, and there was no statistically significant difference between the two groups at 2 days and 12 months after PVP (Table 3).

**Table 3:** Comparison of postoperative VAS, ODI, bone cement leakage, distribution types, recovery, and loss height of injured vertebra between two groups

	Group TPRPA	Group CTPA	Statistics	*P*
VAS				
Preoperative	5.79 ± 0.70	5.82 ± 0.67	*t* = − 0.209	0.835
Postoperative 2 days	2.79 ± 0.96	2.59 ± 0.99	*t* = 0.874	0.385
Postoperative 12 months	0.92 ± 0.85	0.94 ± 0.85	*t* = − 0.100	0.920
ODI				
Preoperative	79.30 ± 10.40	78.04 ± 9.80	*t* = 0.527	0.600
Postoperative 2 days	35.26 ± 3.53	35.21 ± 3.28	*t* = 0.064	0.949
Postoperative 12 months	16.45 ± 5.89	16.47 ± 5.84	*t* = − 0.017	0.987
Bone cement leakage	8(21.05%)	11(32.35%)	*χ*2 = 1.180	0.277
Distribution types				
T1	26 (68.42%)	15(44.12%)	*χ*2 = 4.323	0.038
T2	12	19		
Recovery height of injured vertebra (mm)	3.16 ± 1.76	2.53 ± 1.74	*t* = 1.529	0.131
Lost height of injured vertebra (mm)	0.66 ± 0.67	1.03 ± 0.76	*t* = − 2.227	0.029

### Radiological results

In terms of the bone cement leakage rate and the recovery height of the injured vertebra, there was no significant difference between the two groups (*P* > 0.05). However, there was a significant difference in the distribution of bone cement and the lost height of the injured vertebra between the two groups. In group TPRPA, 8 cases (21.05%) had cement leakage, and 26 cases (68.42%) belonged to type 1. While in the CTPA group, 11 cases (32.35%) had cement leakage and 15 cases (44.12%) belonged to type 2 (Table 3).

## Discussion

Unilateral and bilateral puncture routes are commonly used in the treatment of OLCF by PVP, but there is no consensus on the choice between these two surgical approaches. With the application of G-arm X-ray machine in clinical practice, the difference between bilateral puncture and unilateral puncture in operation time and the amount of radiation exposure is diminishing. However, for hospitals lacking advanced equipment, the choice of surgical method is still an aspect they need to consider. Under the condition of only access to an ordinary C-arm, the unilateral approach has a shorter operation time and a lower radiation dose than the bilateral process [10, 11], which is more acceptable.

The puncture routes of PVP mainly include the conventional transpedicular approach, extrapedicular approach, and transverse process root-pedicle approach. The conventional unilateral transpedicular approach is a common and safe puncture route for PVP and PKP, and it has been used in clinical practice for decades. However, numerous associated complications and problems have been reported, such as puncture difficulty, pedicle fracture, cement leakage, and asymmetric cement distribution [12, 13]. Therefore, some surgeons have used the extrapedicular approach and TPRPA in clinical practice, but the extrapedicular approach is generally only used in cases with mid-thoracic vertebra or a thin pedicle due to the risk of vascular injury [14–18]. Yan L et al. reported TPRPA to be a relatively safe and effective approach with less radiation exposure and a shorter surgical time [5, 19].

In this study, although no specific comparative analysis was conducted on the number and time of X-ray fluoroscopy between the two groups, the total surgical time of the TPRPA group was shorter than that of the CTPA group. As we know, PVP requires fluoroscopy for monitoring throughout the procedure, so we can speculate that the number of X-ray fluoroscopies in the TPRPA group was lower than that in the CTPA group. Of course, this result requires the establishment of the above indicators for rigorous comparative analysis. We believe that TPRPA has an advantage in operation time because of the following: (1) TPRPA insertion point can be explored through anatomical markers, which is more convenient to find the insertion point, while CTPA is more dependent on fluoroscopy to determine the insertion point; (2) the safe puncture range is larger, the success rate of a targeted puncture is higher [8, 20], and there is less chance to adjust the puncture direction due to the puncture angle is not ideal during the operation. All patients in the TPRPA group had no intraoperative pedicle fracture or difficulty in puncture, so we also believe that this technique is safe and reliable when applied to the lumbar spine, which is basically consistent with previous studies.

The VAS and ODI score are important indicators to evaluate the pain degree and quality of life of the patient. Therefore, they are commonly used for evaluating the clinical efficacy before and after treatment. In this study, we compared the clinical effectiveness between the CTPA and TPRPA groups through VAS and ODI. Our study showed that postoperative pain symptoms were significantly relieved, and the quality of life was significantly improved. These results were embodied in the significant changes in the VAS and ODI scores. These results support both CTPA and TPRPA PVP being effective methods for OLCF. In addition, the injection amount of bone cement was different between the two groups, but there was no difference in the therapeutic effect. This result suggests that the clinical effect of PVP is independent of the cement volume.

To our knowledge, many researchers have studied the relationship between bone cement distribution patterns, bone cement leakage, and the therapeutic effect. Bin et al. [21]. found that all cement distribution patterns can relieve pain and reduce the spinal biological curvature, but its extensive distribution to the treated vertebrae has certain advantages in long-term pain relief. Lei et al. [9] proved that sufficient contact of the bone cement with the upper and lower endplates is an ideal distribution type, which can better maintain the height of the fractured vertebra and reduce the risk of long-term vertebral refracture of the vertebral body [22]. Therefore, lateral radiographs were used in this study to evaluate the distribution type of the bone cement and to compare the ratio of the type 1 distribution between the two groups. However, several factors were found to influence the bone cement distribution during PVP procedures, such as uneven bone density, fracture classification, and injection techniques.

In our opinion, injection technology is a factor that the surgeon can control. The puncture point of CTPA goes through the articular process and is close to the inner wall of the pedicle, but the extraversion angle is not sufficient. In contrast, the TPRPA puncture point is more lateral to the facet joint, with a larger extraversion angle, and it is more likely to reach the optimal target position of the anterior and middle 1/3 of the vertebral body. Therefore, the tip of the TPRPA is more likely to reach or exceed the midline.

In this study, compared with the CTPA group, the TPRPA group had more bone cement injected, more type 1 distribution, and less loss of height of the injured vertebra. This result indicated that the injection amount of bone cement was correlated with its distribution, and the distribution type of the bone cement was related to the height loss of the injured vertebra. We hold the point that in the absence of bone cement leakage, increasing the amount of bone cement injection can improve the injection pressure and promote the distribution of the bone cement, and the ideal distribution of the bone cement is beneficial for maintaining the height of the surgical vertebra. In addition, the speed of injection is also a favorable factor to promote the diffusion of bone cement because it can also increase the instantaneous injection pressure.

In this study, a total of 19 cases of bone cement leakage occurred in both groups, all of which were of the disc space and paravertebral area type, without symptoms and not requiring any special treatment. The incidence in the two groups was 21.05%(8/38) and 32.35%(11/34), respectively, but the difference was not statistically significant. The results showed that increasing the volume of the bone cement injection could promote the distribution of bone cement while not increasing the leakage rate of bone cement. However, using an excessive volume of bone cement to obtain an extensive distribution of bone cement is not an ideal method because research based on laboratory biomechanics has found that when the amount of bone cement reaches about 15% of the vertebral body, the stiffness of the damaged vertebral body can be restored. If the amount of bone cement injected exceeds this value, there is no obvious benefit, and it may result in an asymmetric distribution of bone cement and excessive vertebral stiffness [23]. In addition, increasing the amount of bone cement may increase the risk of cement leakage [10, 24]. Although the exact relationship between the cement amount and the cement leakage rate cannot be obtained in this study, we agree with the above viewpoints.

The previous literature reported that the recompression rate of the vertebral body after PVP was 2.9~27.6% [25, 26]. Low bone mineral density is generally recognized as the most important risk factor for recurrent fractures. Other studies had suggested that over time, the bone mass is further reduced, leading to systemic osteoporosis pain [27]. This may lead to rebound pain symptoms during the postoperative follow-up. Therefore, the orthopedist pays more and more attention to anti-osteoporosis treatment after osteoporotic vertebral compression fractures in recent years. Bawa et al [28]. conducted a large sample clinical trial, and the results showed that anti-osteoporosis treatment after fracture could reduce the risk of refracture by 40% compared with patients who did not receive this treatment. In addition, the Clinician’s Guide for the Prevention and Treatment of Osteoporosis recommends that effective anti-osteoporosis therapy is necessary to reduce the risk of additional fractures after the first fracture [29].

As a first-line anti-osteoporosis drug, bisphosphonate is the preferred clinical anti-osteoporosis drug. It directly inhibits the bone resorptive activity of osteoclasts, thus inhibiting bone metabolism and reducing the risk of bone loss to maintain the bone mass and strength and reduce the risk of fractures [30]. We routinely administered bisphosphonates after OLCF. In this study, there was no recurrence of fracture in the two groups during the follow-up period of 12 months, which may be related to the importance we attach to anti-osteoporosis treatment after surgery. However, this conclusion needs to be further studied by extending the follow-up time and setting a control group.

## Limitations

The study has some limitations: (1) Due to the strict inclusion criteria in our study, the number of patients was relatively small; (2) The classification of the distribution type of bone cement using lateral radiographs only, the fine structure was not clearly observed; (3) Although the height of recovery and loss of the operative vertebral were observed, the follow-up time was short, and the evaluation was not comprehensive, especially in term of re-fracture after surgery.

## Conclusions

This study confirmed that both TPRPA and CTPA are effective and feasible used for PVP in the treatment of OLCF. Both methods achieved good clinical outcomes during 12 months of follow-up. However, bone cement was more widely distributed in the vertebral body through TPRPA, which took less operation time without increasing the incidence of bone cement leakage. Moreover, TPRPA is more effective in maintaining the height of the operative vertebra.

## Data Availability

All data are fully available without restriction.

## Abbreviations

PVP: Percutaneous vertebroplasty
OLCFs: Osteoporotic lumbar compression fractures
CTPA: Conventional transpedicular approach
TPRPA: Transverse process root-pedicle approach
VAS: Visual analogue scale
ODI: Oswestry disability index
BMD: Bone mineral density
CT: Computed tomography
MRI: Magnetic resonance imaging
